# Methods to Predict and Lower the Risk of Prostate Cancer

**DOI:** 10.1100/tsw.2011.72

**Published:** 2011-04-05

**Authors:** Barbara Ercole, Dipen J. Parekh

**Affiliations:** Department of Urology, University of Texas Health Science Center, San Antonio, USA

**Keywords:** prostate cancer, chemoprevention, PCPT risk calculator, finasteride, dutasteride

## Abstract

Chemoprevention for prostate cancer (PCa) continues to generate interest from both physicians and the patient population. The goal of chemoprevention is to stop the malignant transformation of prostate cells into cancer. Multiple studies on different substances ranging from supplements to medical therapy have been undertaken. Thus far, only the studies on 5α-reductase inhibitors (the Prostate Cancer Prevention Trial [PCPT] and Reduction by Dutasteride of Prostate Cancer Events [REDUCE] trial) have demonstrated a reduction in the risk of PCa, while results from the Selenium and Vitamin E Cancer Prevention Trial (SELECT) concluded no decreased risk for PCa with selenium or vitamin E.

## INTRODUCTION

The National Cancer Institute estimated that approximately 217,730 new cases of prostate cancer (PCa) would be diagnosed in 2010 and there would be approximately 32,050 PCa deaths[1]. It is the second most common cancer in American men with a 1 in 6 lifetime risk of developing it. The majority of screen-detected PCa is localized, and a concern for overdetection and subsequent overtreatment has been raised. The implication of overtreatment of clinically insignificant PCa is that it puts a large number of men at risk for unnecessary morbidities (urinary, gastrointestinal, sexual) from aggressive treatment of PCa, as well as an unnecessary cost burden on health care. An estimated $8 billion is spent on PCa treatment each year in the U.S.[2] and some have argued that the treatment modalities used for cure in indolent disease is more expensive than those used in treatment of late-stage disease[3]. As such, a risk assessment strategy or chemoprevention strategy may help to decrease these parameters. Mortality from PCa has also decreased over the years, although the direct cause of this is unknown and may be multifactorially related to screening, improved treatment, and chemoprevention. In order to address the impact of screening on population mortality, two large-scale studies, one in the U.S. and one in Europe, attempted to do so in 2009. However, they unfortunately resulted in conflicting conclusions. The Prostate, Lung, Colon, Ovarian, Cancer Screening Project (PLCO) trial in the U.S. randomized men to a fixed period of repetitive prostate specific antigen (PSA) screening visits vs. general community practice. The European Study of Screening for Prostate Cancer (ERSPC) was a similar trial that examined screening in Europe[4,5]. The U.S. trial found no reduction in PCa mortality, while the ERSPC study found a 20% PCa-specific mortality (PCSM) reduction with screening. Both studies had serious limitations. The U.S.

trial was criticized for significant contamination of screening in the control arm, a relatively low rate of biopsy in those men with abnormal screening results, and a short follow-up period. The ERSPC trial has also been criticized, mainly due to its wide heterogeneity of study designs. The mortality reduction was achieved as a result of an investment in a large amount of resources and time; to prevent one PCa death, over 1400 men required screening over almost a decade and almost 50 men required treatment. These studies have been recently reanalyzed. Crawford et al.[6] reanalyzed the data for the PLCO trial by using different comorbidity strata to evaluate estimates of PCSM. In men with no or minimal comorbidity, annual screening was associated with a significant reduction in the risk of PCSM. These men were also more likely to undergo curative therapy than those men with significant comorbidities. As the authors pointed out, these findings are hypothesis generating as the analysis was performed postrandomization. The 35.7% estimate of men with no or minimal comorbidities may be an overestimate when applied to the general population. Also, the number needed to treat (NNT) was adjusted to 5; thus, both overdetection and overtreatment are still a risk. Loeb et al.[7] reanalyzed the data from the ERSPC trial by analyzing the effects of varying follow-up times on number needed to screen (NNS) and NNT, as these are time-specific variables and quoting a single time point may be misleading. They found that at 9 years, the NNS was 1,254 and NNT was 43. These variables continued to decrease with each subsequent analysis; at 10 years, NNS decreased to 837 and at 12 years to 503. NNT decreased to 29 at 10 years and to 18 at 12 years. Of note, as the number of years increases, the authors point out that the differences in the mortality between the two arms will continue to grow and, therefore, the NNT estimates will continue to decrease.

Another randomized, population-based screening trial recently reported its first planned analysis on mortality outcomes[8]. The Goteborg trial randomized 20,000 men to either screening with PSA every 2 years (n = 10,000) vs. a control group (n = 10,000). The primary end point was PCSM and the study was analyzed according to an intention-to-screen principle. Median age of participants was 65 and prostate biopsies were performed if PSA was 3.4 ng/mL during 1995–1998, and subsequently lowered to 2.5 ng/mL after 2005. During a median follow-up of 14 years, they have thus far noted a 12.7% PCa incidence in the screening group compared to 8.2% in the control group. The cumulative relative risk reduction of death was 50% in the screening group, and they have thus far determined that NNS was 293 and NNT was 12. This report has only studied the early effects of screening, as this is a young population (median age 65), and has a comparatively short follow-up time after PCa diagnosis. The relevant reduction in cancer mortality found in this study is comparable to that with breast or colorectal cancer screening, although the authors warn that varying lead times may result in the risk of overdiagnosis. Even though these studies are helping to shed light on PSA screening, the warning of overdetection and overtreatment are a recurring theme and, thus, screening alone might not be sufficient to prevent PCa morbidity and mortality. While several risk prediction models for PCa detection are available, we highlight the Prostate Cancer Prevention Trial (PCPT) risk calculator for the purpose of this review since it is the only model derived from a phase III prospective randomized trial.

## PCPT RISK CALCULATOR

The Southwest Oncology Group (SWOG) supported by the National Cancer Institute (NCI) released the results of the PCPT in 2003 under recommendations from the Data Safety and Monitoring Committee[9]. The PCPT risk calculator was derived from the placebo arm of the PCPT trial[10]. A total of 5519 subjects in the placebo group who had a PSA test and digital rectal exam (DRE) within 1 year of biopsy were selected for development of the calculator. This group of men was predominantly Caucasian and included men who were ≥55 years, with a normal DRE, and a PSA ≤3.0 ng/mL. In a multivariable analysis, variables that had a statistically significant association for an increased risk of PCa included increasing log PSA (odds ratio [OR] = 2.34, $p < 0.001$), positive family history of PCa (OR = 1.31, $p = 0.002$), and abnormal DRE result (OR = 2.47, $p < 0.001$). A statistically significant decreased risk of PCa was noted with a previous history of one or more negative biopsies (OR = 0.64, 95% CI = 0.53 to 0.78, $p < 0.001$). Statistically significant predictors of high-grade disease included PSA level (OR = 3.64, $p <$

0.001), an abnormal DRE (OR = 2.72, $p < 0.001$), and race, where African American men had a higher risk of high-grade disease than non–African Americans (OR = 2.61, $p < 0.001$). Even though age was statistically significant as associated with increased risk of high-grade disease, each incremental 1 year in age had an OR of 1.03 ($p = 0.54$). A decrease risk in PCa was again noted with a previous history of one or more negative biopsies (OR = 0.70, $p = 0.04$). The PCPT risk calculator was developed using these variables and is available on-line[11]. This tool is applicable to men age 55 or older who do not have a diagnosis of PCa and are undergoing a prostate biopsy. The results obtained after entering the above variables result in a percentage risk of PCa and high-risk PCa on prostate biopsy. The PCPT risk calculator has been validated in a number of studies since 2006[12,13,14]. Recently, the risk calculator was updated to include PCA3 measurements[15], body mass index, and whether or not the patient is on finasteride. Certainly, limitations exist with the calculator. One such limitation is the dichotomous variable for prior biopsies; recent publications noted that an increase in the number of negative biopsies predicts a decrease in the risk of PCa detection[16]. The calculator is a work in progress, and is being constantly updated and validated. The value in having a tool such as the PCPT risk calculator is that it individualizes the risk for detecting PCa in a biopsy based on the patients' variables; therefore allowing the patients to determine their personalized level of risk, not just by PSA alone, but through a preliminary assessment that would lead to a prostate biopsy.

## RATIONALE FOR PREVENTION

Prevention strategies for PCa may have two goals: one to prevent disease and the other to modulate the risk of progression of premalignant lesions. Either one is beneficial in that a man would not have to deal with a diagnosis of PCa. A prevention strategy should have minimal toxicity, be inexpensive, and have a reasonably good ability to reduce the risk of disease. Prevention strategies have been utilized successfully in other diseases and malignancies, such as in cardiovascular disease and breast cancer[17,18]. As with treatment options, preventive strategies could carry a high economic burden as well as quality of life–related issues; however, the burden of treatment-related morbidity, such as incontinence and impotence, for PCa is not an issue.

## CHEMOPREVENTION

Testosterone, which is primarily produced by Leydig cells, is converted to dihydrotestosterone (DHT) within prostate cells by 5α-reductase type I and II enzymes. Type I 5α-reductase is the predominant isoform in PCa tissue and, to a lesser extent, in benign prostatic hyperplasia (BPH) tissue, while type II is present in normal prostatic tissue and BPH[19,20]. There is strong evidence indicating an association between these two androgens and the risk of PCa. Modulation of the production of DHT has been studied at the level of the 5α-reductase type I and II enzymes. DHT has a greater affinity for the androgen receptor in prostate tissue than testosterone does and plays a crucial role in the development of the gland. It is well established that individuals with a hereditary deficiency in these enzymes do not have appropriate development of their prostate and external genitalia[21].

### Finasteride

Finasteride is a competitive inhibitor of the type II 5α-reductase enzyme within prostatic cells[22]. Extensive evidence from preclinical data and clinical trials have indicated or demonstrated a role for 5α-reductase inhibitors in the treatment of BPH, prevention of BPH-related outcomes, as well as prevention of PCa[23,24,25]. Data from preliminary trials helped to support the notion that finasteride might be effective in the prevention of PCa. The PCPT was the first phase III trial conducted for the prevention of

PCa. This study found a 24.8% ($p < 0.001$) relative risk reduction in the prevalence of PCa over its 7-year period in men who received finasteride. Since its publication, multiple reports have clarified the concerns and effects of finasteride on PCa. Finasteride significantly enhances the ability of PSA to detect PCa and high-grade PCa, increasing sensitivity and specificity of this biomarker when compared to PSA's performance in the placebo group. The area under the curve (AUC) for PSA was increased from 0.681 to 0.757 ($p < 0.001$); for Gleason 7-10 disease, it was increased from 0.781 to 0.838 ($p = 0.003$); for Gleason 8-10 disease, it was increased from 0.824 to 0.886 ($p = 0.071$)[26]. The effects of finasteride on DRE demonstrated an increase in sensitivity of DRE in detecting PCa (16.7 to 21.3%; $p = 0.015$) with no reduction in its specificity and in regards to the detection of high-grade disease, it was noted that there was an increase in sensitivity, but this did not reach statistical significance[27]. A reduction from 11.7 to 9.2% ($p < 0.001$) was noted when the impact of finasteride on the risk of development of high-grade prostatic intraepithelial neoplasia was evaluated[28]. An improvement in the detection of high-grade PCa was also confirmed. When evaluating the prostatectomy specimen in those men who underwent a radical prostatectomy in the placebo group, it was observed that about 50% of the men who were ultimately found to have high-grade PCa (Gleason 7-10) in the radical prostatectomy specimen had low-grade cancer (Gleason ≤ 6) found at the time of biopsy. This rate of undergrading was reduced to about 30% in the finasteride group. This outcome is not fully understood at this time; however, a reasonable explanation is the 25% reduction in gland volume in the finasteride group that leads to a more accurate sampling of the gland at time of biopsy. Independent analysis of the PCPT data has confirmed the increase in high-grade detection of PCa in the finasteride arm to be due to detection and sampling biases, as well as improvement in the performance characteristics of prostate biopsy[29,30,31,32].

## Dutasteride

Dutasteride is a type I and II 5α-reductase inhibitor that reduces serum DHT by more than 90%[33,34]. Its effect on chemoprevention for PCa was tested in the REDUCE (Reduction by Dutasteride of Prostate Cancer Events) trial[35]. This study design was based on the results of the PCPT as well as preliminary results with dutasteride. In a randomized trial of dutasteride for BPH (n = 4325), Andriole et al. demonstrated an approximate 50% reduction in PCa in the dutasteride group at 27 months (2.5 to 1.2%; $p = 0.002$)[36]. The REDUCE trial was subsequently designed and powered to further test these findings in two randomized arms: dutasteride 0.5 mg daily vs. placebo[37]. Unlike the PCPT, this study enrolled men who were at high risk for developing PCa based on age, PSA level, and previous suspicion of PCa that led to a biopsy. Men in the 50–60 age range were eligible to participate if they had a PSA within 2.5–10 ng/mL; for those men in the 60–75 age range, their PSA level had to be between 3–10 ng/mL. Participants who had undergone a single biopsy within 6 months prior to enrollment and were negative were also eligible. This study was a randomized, multicenter, double-blind, placebo- controlled, parallel-group study conducted for 4 years after a 4-week run-in period of placebo. Biopsies were obtained at 2 and 4 years. Cancer was detected in 659 of the 3305 men in the dutasteride group compared to 858 of the 3424 men in the placebo group, equating to a relative risk reduction with dutasteride of 22.8% (95% CI 15.2–29.8; $p \leq 0.001$) in 4 years[35]. There was no statistically significant increase in incidence of high-grade disease in the dutasteride arm. In regards to BPH end points, dutasteride showed a positive effect in the reduction of symptoms, mainly episodes of urinary retention.

Although a risk reduction in PCa incidence has been noted with the use of finasteride and dutasteride, these drugs are currently not FDA approved for the use as chemopreventive agents.

## SELECT Trial

The Selenium and Vitamin E Cancer Prevention Trial (SELECT) randomized 35,533 men to four arms: selenium (200 µg/day), vitamin E (400 IU/day), both, or placebo[38]. There were no statistically significant differences noted between the groups on decreasing PCa risk. Although the results did not reach statistical significance, there was an increased risk of PCa in the vitamin E group ($p$ = 0.06) and of type 2 diabetes mellitus in the selenium group (relative risk, 1.07; 99% CI, 0.94–1.22; $p$ = 0.16), but not in the selenium and vitamin E group. The conclusion from this study was that neither selenium nor vitamin E helped to prevent PCa at the doses administered.

## CONCLUSION

Continued efforts to determine the best methods to predict and lower the risk of PCa are ongoing as there is growing concern for the current overdetection and overtreatment of PCa with PSA screening. Chemoprevention is a concept worth pursuing as this strategy has proved useful in other disease processes, such as cardiovascular disease and diabetes.
